# First Report on the Plasmidome From a High-Altitude Lake of the Andean Puna

**DOI:** 10.3389/fmicb.2020.01343

**Published:** 2020-06-23

**Authors:** María Florencia Perez, Daniel Kurth, María Eugenia Farías, Mariana Noelia Soria, Genis Andrés Castillo Villamizar, Anja Poehlein, Rolf Daniel, Julián Rafael Dib

**Affiliations:** ^1^Planta Piloto de Procesos Industriales Microbiológicos, Consejo Nacional de Investigaciones Científicas y Técnicas, San Miguel de Tucumán, Argentina; ^2^Genomic and Applied Microbiology & Göttingen Genomics Laboratory, Institute of Microbiology and Genetics, Georg-August University of Göttingen, Göttingen, Germany; ^3^Línea Tecnológica Biocorrosión, Corporación para la Investigación de la Corrosión C.I.C., Piedecuesta, Colombia; ^4^Facultad de Bioquímica, Química y Farmacia, Instituto de Microbiología, Universidad Nacional de Tucumán, San Miguel de Tucumán, Argentina

**Keywords:** plasmidome, extreme environment, resistance, high-altitude lake, Andean Puna, functional profile

## Abstract

Mobile genetic elements, including plasmids, drive the evolution of prokaryotic genomes through the horizontal transfer of genes allowing genetic exchange between bacteria. Moreover, plasmids carry accessory genes, which encode functions that may offer an advantage to the host. Thus, it is expected that in a certain ecological niche, plasmids are enriched in accessory functions, which are important for their hosts to proliferate in that niche. Puquio de Campo Naranja is a high-altitude lake from the Andean Puna exposed to multiple extreme conditions, including high UV radiation, alkalinity, high concentrations of arsenic, heavy metals, dissolved salts, high thermal amplitude and low O_2_ pressure. Microorganisms living in this lake need to develop efficient mechanisms and strategies to cope under these conditions. The aim of this study was to characterize the plasmidome of microbialites from Puquio de Campo Naranja, and identify potential hosts and encoded functions using a deep-sequencing approach. The potential ecological impact of the plasmidome, including plasmids from cultivable and non-cultivable microorganisms, is described for the first time in a lake representing an extreme environment of the Puna. This study showed that the recovered genetic information for the plasmidome was novel in comparison to the metagenome derived from the same environment. The study of the total plasmid population allowed the identification of genetic features typically encoded by plasmids, such as resistance and virulence factors. The resistance genes comprised resistances to heavy metals, antibiotics and stress factors. These results highlight the key role of plasmids for their hosts and impact of extrachromosomal elements to thrive in a certain ecological niche.

## Introduction

Horizontal gene transfer contributes significantly to both plasticity and evolution of prokaryotic genomes (Koonin and Wolf, 2008), and mobile genetic elements act as drivers of these processes (Frost et al., 2005). Among such elements, plasmids are considered important vectors in genetic exchange between bacteria (Halary et al., 2010). It is also thought that plasmids, which are abundant in many environments, confer important ecological functions in each niche (Heuer et al., 2008). Plasmids often encode DNA replication and mobilization genes that ensure their maintenance within the host and transfer from one host to another. Moreover, plasmids carry accessory genes which encode functions that usually are selected by evolutionary forces. Intake of plasmids offers an advantage for the host by gaining, e.g., virulence factors, resistance to antibiotics, production of antimicrobials, degradation of xenobiotics, and other traits (Novick, 2003; Crossman, 2005; Gil et al., 2006; Heuer et al., 2008; Kav et al., 2012). Therefore, it is expected that in a certain ecological niche, plasmids are enriched in accessory functions, which are important for their hosts to thrive in that niche. Plasmids are considered as key elements in the study of microbial habitats, and to characterize them as a whole is necessary to understand the functioning of microbial environments.

To date only a few studies targeting the plasmidome (overall plasmid population, also known as plasmid metagenome or metaplasmidome) of microbial ecosystems using culture-independent methods have been published. Kav et al. (2012) have been pioneers in studying the composition and importance of plasmids in cow rumens. Studies from the activated sludge plasmid metagenome of wastewater treatment plants were also conducted, in which genes for carbohydrate metabolism as well as antibiotic and heavy metal resistance genes were abundant (Zhang et al., 2011; Sentchilo et al., 2013). Recently, Kothari et al. (2019) analyzed the plasmidome of groundwater samples, reporting plasmids highly enriched in metal resistance.

In extreme environments such as Andean lakes in which microbialites and microbial mats are commonly found, studies on the entire mobilome have not been reported yet. Microbialites are organo-sedimentary deposits accreted by sediment trapping, binding and *in situ* precipitation due to the growth and metabolic activities of microorganisms (Burne and Moore, 1987). We have previously shown that these environments harbor diverse extremophilic microorganisms (Dib et al., 2009; Ordoñez et al., 2009; Farías et al., 2013, 2014; Rasuk et al., 2014, 2016; Albarracín et al., 2015, 2016). These microorganisms have evolved efficient mechanisms and strategies that allow them to survive under extreme conditions. Such conditions include elevated UV radiation, alkalinity, high concentrations of arsenic, heavy metals and dissolved salts, low O_2_ pressure and high thermal amplitude. Among the isolated respective bacterial strains, actinobacteria resistant to high UV radiation, arsenic and different antibiotics have been found (Dib et al., 2008, 2009). Furthermore, it has been observed that they carried giant linear plasmids, encoding genes required to thrive under harsh environmental conditions (Dib et al., 2010a, b, 2013a,b, 2013c). Sequencing and analysis of these genetic elements revealed the presence of genes encoding resistance to UV radiation, antibiotics and oxidative stress, indicating that many adaptive factors were encoded at the extrachromosomal level (Dib et al., 2013b, c, 2015, 2018).

**FIGURE 1:**
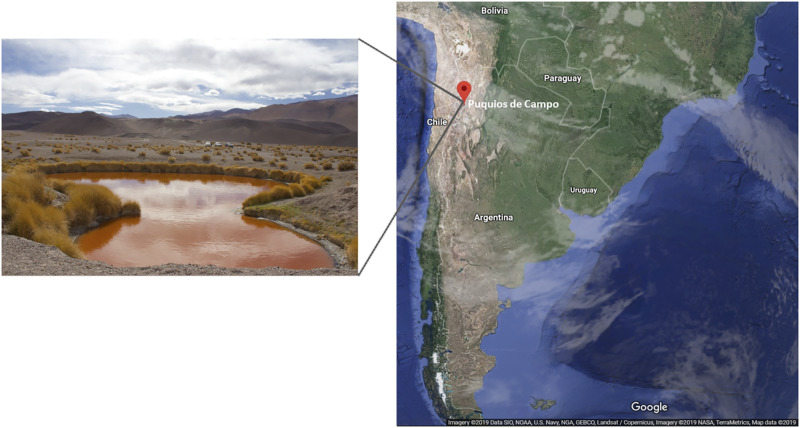
Puquio de Campo Naranja lake, located in Catamarca province, Argentina.

Puquios de Campo is located in the Salar de Antofalla, at 3,340 m above sea level, in the northwest of Catamarca province, Argentina. Among eight small natural pools (around 40 m diameter), Puquio de Campo Naranja is exceptional due to its intense red color (Figure 1). Microorganisms living in this location face multiple extreme conditions under which plasmids might play significant roles in adaptive as well as in evolutionary processes. Besides a few studies on plasmids from cultivable bacteria, the global plasmid community in these environments has not been studied. The aim of this study was to characterize the plasmidome of microbialites from Puquio de Campo Naranja, and identify potential hosts and encoded functions using a deep-sequencing-based metagenomic approach.

## Materials and Methods

### Sampling and Biomass Purification

Samples were taken from microbialites of Puquio de Campo Naranja, Catamarca, Argentina (25°36′38.05″S, 67°40’20.95”W) in May of 2017. Soil analysis of the sampling site revealed a high salinity and metal concentrations (Supplementary Table S1). Permission for sample collection was granted by the Secretaría de Medio Ambiente, Catamarca, Argentina (No. 22935/2016).

Microbialites were found in a distance of 10 cm from the lake shore. Triplicate cores (3 cm^2^ each) including several mat layers were taken to a depth of 3 cm and pooled in order to obtain representative samples. Then, they were disaggregated, placed in sterile plastic flasks, stored at 4°C and processed within a week.

Microorganisms were separated from the sample by using the following protocol: 40 mL of 10% w/v NaCl were added to 15 g of sample and incubated at 40°C for 15 min to dissolve extracellular polymeric substances (EPS) (Farías et al., 2013). Subsequently, it was centrifuged at 4°C and 7,100 × *g* for 15 min (UNIVERSAL 320 R centrifuge, Hettich, Germany) and the pellet was resuspended in 15 mL of 4X PBS buffer (stock solution 10X: 80 g L^–1^ NaCl, 2 g L^–1^ KCl, 14.4 g L^–1^ Na_2_HPO_4_, 2.4 g L^–1^ KH_2_PO_4_, pH 7.4). After subjecting the sample to an ultrasonic water bath for 15 min (TB 010 TA, Testlab, Argentina), cells were collected by gentle centrifugation at 4°C and 700 × *g* for 1 min (UNIVERSAL 320 R centrifuge). This last procedure with 4X PBS buffer was repeated three times and the resulting supernatants were pooled and centrifuged at 4°C and 7,100 × *g* for 15 min (UNIVERSAL 320 R centrifuge). Biomass purification was performed twice to obtain two pellets, which were kept at -20°C until plasmid DNA extraction.

### Plasmid DNA Extraction

The total plasmid DNA was isolated by two different methods in order to maximize the obtained yield. In the first protocol (Kado and Liu, 1981), the pellet was resuspended in 35 mL of lysis buffer (3% w/v SDS, 50 mM TRIS, pH 12.6) and incubated at 60°C for 1 h. Then it was extracted twice with 1 volume of phenol/chloroform solution (1:1, v/v) and the aqueous phase was separated by centrifugation at 4°C and 23,600 × *g* for 15 min (Sorvall RC-5C, SS-34 rotor, DuPont, United States). After two extractions with 1 volume of chloroform, the aqueous phase was transferred to a new tube and DNA precipitation was carried out by adding 0.6 volume of isopropanol and 0.1 volume of 3M sodium acetate (pH 5.2), followed by incubation overnight at 4°C. The plasmid DNA pellet was obtained after centrifugation at 4°C and 23,600 × *g* for 30 min (Sorvall RC-5C, SS-34 rotor, DuPont, United States). The recovered pellet was washed twice with 70% ethanol. Finally, DNA was dried and resuspended in 400 μL of double-distilled water.

A second extraction procedure was performed following the protocol of Hansen and Olsen (1978). Cells were suspended in 16 mL of a 25% w/v sucrose and 50 mM TRIS buffer (pH 8). Subsequently, 120 μL of lysozyme (50 mg mL^–1^; USB Corporation, Cleveland, OH United States) were added. The suspended cells were mixed by inversion and incubated on ice for 5 min. Next, 3 mL of 250 mM EDTA (pH 8) were added and the cells were mixed by inversion and incubated on ice for 5 min. Subsequently, 3 mL of a 20% SDS in TE buffer were also added and mixed by inversion. Eight cycles of heat and mixing were carried out (one cycle includes 15 s at 55°C and five inversions during 15 s at room temperature). Then, 3 mL of 3M NaOH were added to denature the DNA. After 3 min of inversion, the mixture was neutralized with 6 mL of 2M TRIS (pH 7). Subsequently, 4 mL of a 20% SDS in TE buffer and 7.5 mL of 5M NaCl were added to the mixture and cooled on ice. After overnight incubation at 4°C and centrifugation at 4°C and 9,200 × *g* for 30 min (Sorvall RC-5C, SS-34 rotor, DuPont, United States), the supernatant containing the plasmid DNA was precipitated and washed as described above.

In parallel, samples from Puquio de Campo Naranja were also used for metagenomic DNA extraction using FastDNA^TM^ SPIN kit for soil as recommended by the manufacturer (MP Biomedicals, United States). The generated dataset from sequencing was used to compare the deduced functional profile with the plasmidome dataset.

### Chromosomal DNA Removal

The plasmid DNA from both methods was pooled and subjected to overnight digestion at 37°C with exonuclease V Rec BCD (New England Biolabs, Ipswich, Massachusetts, United States) to remove chromosomal DNA. To test chromosomal DNA contamination PCR reactions using universal primers including the V4 region of 16S rRNA gene were performed. The primers used for bacteria were F5′-CCTACGGGNGGCWGCAG-3′ and R5′-GGATTAGATACCCBDGTAGTC-3′ (Klindworth et al., 2013); and for archaea F5′-CCCTAYGGGGYGCASCAG-3′ and R5′-ATTAGAKACCCSNGTAGTCC-3′ (Gantner et al., 2011). The plasmid DNA was purified using SureClean Plus kit as recommended by the manufacturer (BIOLINE, London, United Kingdom). Subsequently, concentration was measured by Qubit fluorometric quantification (Invitrogen, Thermo Fisher Scientific, CA, United States).

### Sequencing, Quality Control, and Assembly

The isolated plasmid DNA was used to generate Illumina shotgun paired-end sequencing libraries. The MiSeq system together with MiSeq reagent kit version 3 was used for sequencing of the plasmidome as recommended by the manufacturer (Illumina, San Diego, CA, United States). The paired-end metagenome sequencing was performed with a Hiseq 2500 system (2 × 250) by using the SBS kit v2 as recommended by the manufacturer (Illumina). The quality control of raw sequence reads was carried out with FastQC tool version 0.11.5 and the reads were quality-filtered using Trimmomatic version 0.36 (Bolger et al., 2014). Reads were *de novo* assembled by using metaSPAdes software version 3.11.0 (Nurk et al., 2017).

### Bioinformatic Analysis

The reads generated by sequencing of the plasmid DNA were aligned with the metagenome contigs of this environment using the Bowtie2 tool (Langmead and Salzberg, 2012). Annotation and labeling of all the relevant genomic characteristics on plasmidome contigs were done with Prokka version 1.11 (Seemann, 2014). The plasmidome dataset was submitted to the MG-RAST server (Meyer et al., 2008) for functional and taxonomic analysis. Comparisons with the SEED subsystem database were performed using a maximum *E*-value of ≤ 10^–5^. The deduced functional profile of the plasmidome from the Puna was compared with the one derived from the corresponding metagenome and with the plasmidome of a wastewater treatment plant (Sentchilo et al., 2013) by employing the software STAMP (Parks and Beiko, 2010).

A database of plasmids was created from all plasmid sequences in NCBI and compared with the plasmidome dataset using BLAST standalone (Altschul et al., 1990). Best hits were selected by following parameters: identity ≥ 90% and query coverage ≥ 90% (hit length at least 100 bp).

Complete translated gene sequences identified with Prokka were searched for domains related to plasmid replication and mobilization using HMMER 3.3 (cut-off *E*-value < 10^–3^), the Pfam database version 32.0 (El-Gebali et al., 2019) and MOBfamDB database (Garcillán-Barcia et al., 2020). Pfam families covering known plasmid replicon domains were selected from Schlüter et al. (2008); Ma et al. (2012), Sentchilo et al. (2013); Jørgensen et al. (2014), Shi et al. (2018) and our own additions.

Putative genes encoding metal resistance and virulence factors were identified by comparisons between plasmidome of Puquio de Campo Naranja and the BacMet (Pal et al., 2014) and VFDB databases (Chen et al., 2005). For this purpose, the generated Prokka annotation file was subjected to Conditional Reciprocal Best BLAST (crb-blast) with an *E*-value of ≤ 10^–3^. Hits with an identity ≥ 70% and an alignment coverage ≥ 90% were selected and their abundances were calculated. The Resistance Gene Identifier (RGI) software was employed for prediction of antibiotic-resistance genes using the Comprehensive Antibiotic Resistance database (CARD) (Jia et al., 2017) as a reference.

All nucleotide sequences from INTEGRALL database (Moura et al., 2009) were downloaded and compared with the plasmidome sequences by blastn and Bowtie2 (Langmead and Salzberg, 2012). The ISfinder platform was used to search for mobile elements such as insertion sequences or transposons (Siguier, 2006).

### Amplicon Sequencing and Phylogenetic Analysis

16S rRNA gene amplicon sequencing using metagenomic DNA as template was performed using the above-described universal primers covering the 16S rRNA gene sequence. The MiSeq system together with MiSeq reagent kit version 3 was used for sequencing of the amplicons as recommended by the manufacturer (Illumina). Data quality control and analysis were performed using the QIIME 1.9 pipeline. First, paired-end reads were joined with PEAR (pear-0.9.10-bin-64). Quality filtering was performed using the split_libraries_fastq. py script. Forward and reverse primers were removed by using cutadapt. USEARCH was used for zero-radius operational taxonomic unit (zOTU) determination. Taxonomy was assigned against Silva 128 database (Yilmaz et al., 2014).

## Results and Discussion

### Isolation and Initial Analysis of the Plasmidome

Plasmid DNA from the microbial community present in the sample derived from Puquio de Campo Naranja was isolated using two different methods. Both were effective to obtain plasmid DNA for further studies. After DNA pooling, exonuclease treatment and purification, the final DNA concentration was 58.6 μg mL^–1^. Sequencing of the plasmidome yielded 7,075,125 high-quality reads. Assembly resulted in 30,545 contigs (>1,000 bp) with a total contig length of approximately 59 Mb, which is considerable larger than in most of the previous reports. Zhang et al. (2011) obtained a total of 4,641 contigs (>500 bp) and total contig length of 7.1 Mb for the plasmidome of activated sludge. Kav et al. (2012) achieved 13,018 and 5,771 contigs for the plasmidome derived from cow rumen using two different assembly programs.

### Functional Profile Deduced From the Plasmidome

A total of 248,147 coding sequences (61%) of the plasmidome comprise predicted proteins with known function and 155,205 (39%) putative proteins without assigned function. Thus, the plasmidome appears as a source of novel genes and genes products. In comparison, Sentchilo et al. (2013) found that only 34 and 48% of the coding sequences derived from two wastewater treatment plant plasmidomes could be functionally annotated.

**FIGURE 2:**
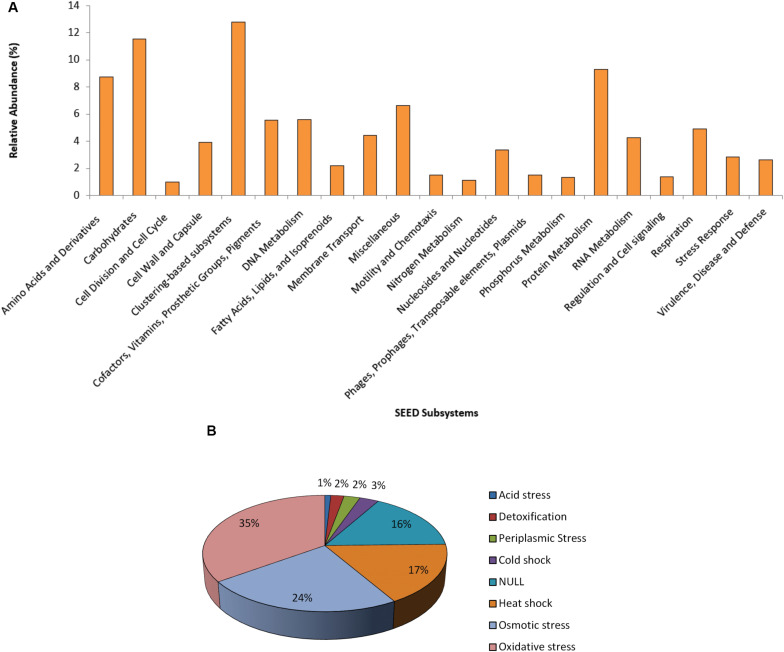
Functional profile of the plasmidome. **(A)** The Puquio de Campo Naranja plasmidome sequences were compared with the SEED database for relative abundance of each of the major SEED subsystems by using a maximum *E*-value of ≤ 10^– 5^. SEED subsystems that were less abundant than 1% are not shown. **(B)** “Stress Response” subsystem level 2 classification of the SEED database. The Puquio de Campo Naranja plasmidome was compared with the SEED database for relative abundance of each category from “Stress Response” subsystem by using a maximum *E*-value of ≤ 10^– 5^. Relative abundance was calculated as the percentage of total matches with the database corresponding to each subsystem or category.

The functional SEED assignment of the Puquio de Campo Naranja plasmidome revealed that the most highly represented subsystems are “Clustering-based subsystems” (12.8%) and “Carbohydrates” (11.5%), followed by “Protein Metabolism” (9.3%) and “Amino Acids and Derivatives” (8.7%). The next most abundant subsystems were “Miscellaneous” (6.6%) and “DNA Metabolism” (5.6%) (Figure 2A). These functional categories were also the most represented in the groundwater plasmidome reported by Kothari et al. (2019). In the “DNA Metabolism” subsystem, approximately 47% of the genes were related to DNA repair functions and 21% to DNA replication functions. The latter included genes specific for plasmid replication. The large proportion of DNA repair functions could be explained through the high UV radiation exposure of microorgansims in the Andean Puna, which may directly and indirectly affect DNA integrity. Repair pathways, apart from protecting cells against the effects of endogenous damage (Dalhus et al., 2009), confer resistance or are an adaptive mechanism to the extreme environment. Accordingly, in previous studies, a set of almost one hundred UV-resistant strains were already isolated and identified in other high-altitude Andean lakes (Fernández Zenoff et al., 2006; Zenoff et al., 2006; Dib et al., 2008; Flores et al., 2009; Ordoñez et al., 2009; Bequer Urbano et al., 2013). In addition, Kurth et al. (2015) encountered DNA repair genes in *Acinetobacter* sp. Ver 3, a strain isolated from a lake of the Andean Puna.

Although less abundant, the subsystems “Stress Response” and “Virulence, Disease and Defense” were also represented in the plasmidome. Genes related to oxidative and osmotic stress responses were mainly present (Figure 2B). In the “Virulence, Disease and Defense” subsystem, approximately 83% genes belonged to the “Resistance to antibiotics and toxic compounds” category. As it has been already mentioned, UV radiation also acts indirectly by producing photo-oxidizing compounds and reactive oxygen species (ROS), such as hydrogen peroxide, superoxide and hydroxyl radicals that damage DNA, proteins, and lipids (Demple, 1994; Wilson et al., 2004). Thus, it is indicated that adaptation to this environment requires the development of mechanisms providing tolerance to UV radiation, e.g., oxidative stress response systems.

**FIGURE 3:**
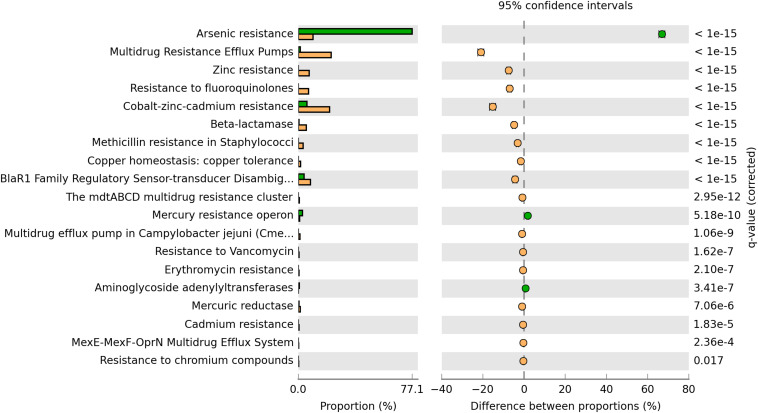
Comparison of the functional profile derived from the Puquio de Campo Naranja plasmidome (orange bars) with the one derived from the wastewater treatment plant plasmidome in Visp, Switzerland (green bars). Percent “Resistance to antibiotics and toxic compounds” categorizable protein coding genes belonging to the “Virulence, Disease and Defense” SEED subsystem, and pairwise proportional differences calculated using STAMP (Statistical Analysis of Metagenomic Profiles). Fisher’s exact test was used and corrected *P*-values were calculated using Benjamini-Hochberg‘s FDR. Only the statistically significant SEED subsystems are shown (*q* < 0.05).

The plasmidome of this high-altitude lake was compared with the plasmidome of a wastewater treatment plant located in Visp, Switzerland (WWTP Visp), which treats a mixture of household wastewater and effluents of the chemical/pharmaceutical industry (Sentchilo et al., 2013). When genes associated with resistances to antibiotics and toxic compounds were analyzed, genes encoding multidrug resistance efflux pumps, Zinc resistance, Cobalt-Zinc-Cadmium resistance as well as resistance to fluoroquinolones and β-lactam antibiotics showed higher relative abundance in the Puquio de Campo Naranja plasmidome than in the WWTP Visp plasmidome (Figure 3). Arsenic resistance-encoding genes were found in higher abundance in the plasmidome from the wastewater treatment plant containing effluents of the chemical/pharmaceutical industry (WWTP Visp), but they were also well represented in the plasmidome of this extreme community, located at a site where a high concentration of arsenic was present (82.2–109 mg Kg^–1^; Supplementary Table S1). No significant differences between plasmidomes in the abundances of other resistance genes were detected, suggesting a certain degree of similarity, despite the differences of a pristine and an anthropogenically-impacted environment (Figure 3).

### Plasmidome vs. Metagenome From Puquio de Campo Naranja

Metagenomic DNA was also extracted from the Puquio de Campo Naranja sample and analyzed to allow comparison with the plasmidome. Only 30% of plasmidome reads aligned with the metagenome-derived contigs. This could be due to the low representation of plasmids during metagenome sequencing of total microbial community DNA (Li et al., 2012). Thus, the plasmids sequences are underrepresented and somehow hidden in the metagenomic DNA. Accordingly, the separation of plasmid DNA and chromosomal DNA before sequencing is important and necessary. It is important to mention that bioinformatic tools for the analysis of plasmids from total metagenomic DNA are limited (Arredondo-Alonso et al., 2017). Furthermore, the possibility of only sequencing the extrachromosomal DNA instead of the full metagenome increases the chance of obtaining the full sequence of particular plasmids.

**FIGURE 4:**
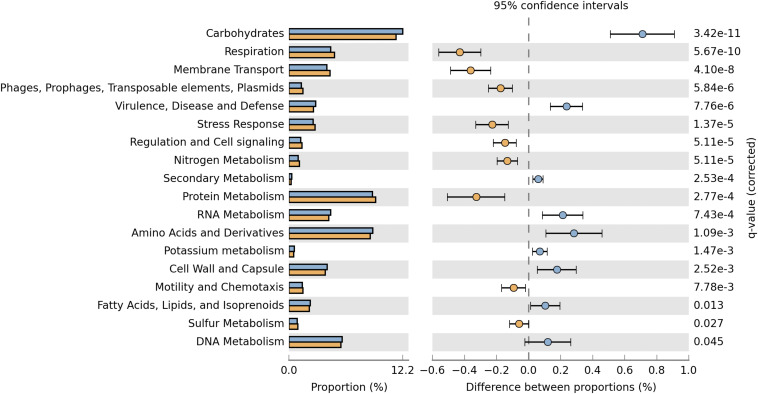
Comparison of the functional profiles derived from the plasmidome (orange bars) and the metagenome (blue bars) of Puquio de Campo Naranja. Percent SEED categorizable protein coding genes and pairwise proportional differences calculated using STAMP. Fisher’s exact test was used and corrected *P*-values were calculated using Storey’s FDR. Only the statistically significant SEED subsystems are shown (*q* < 0.05).

The deduced functional distribution of the plasmidome was further compared with the metagenome by using the datasets derived from MG-RAST and STAMP for statistical analysis. The plasmidome was significantly enriched in genes associated with respiration, membrane transport, regulation and cell signaling, and protein metabolism compared to the metagenome (Figure 4). Genes that are commonly encoded by plasmids, belonging to the “Phages, Prophages, Transposable elements, Plasmids” and “Stress Response” subsystems were also more abundant in the plasmidome. The same was recorded for the “Nitrogen Metabolism” in which genes related to nitrogen fixation are included.

Although the “Virulence, Disease and Defense” subsystem is more enriched in the metagenome, it is also well represented in the plasmidome (Figures 2A, 4), but significant differences between the plasmidome and metagenome in the category “DNA Metabolism,” which contains genes associated to DNA repair, were not detected (Figure 4).

### Plasmidome Matches With Known Plasmids

In this study, plasmid sequences present in the NCBI database were searched in the plasmidome dataset (Table 1). Strict cut-off criteria as sequence identity ≥90% and query coverage ≥90% were adopted (hit length at least 100 bp). Matches were affiliated to plasmids pDSHI01, pDSHI03, and pDSHI04 of *Dinoroseobacter shibae* DFL 12, which was isolated from cultures of marine dinoflagellates derived from the Bay of Tokyo, Japan (Biebl et al., 2005). This marine bacterium is a member of the *Roseobacter* group and represents one model organism for study the life of this group in the ocean. The strain DFL 12 harbors five plasmids, which carry 628 kb of extrachromosomal information, including a *vir* gene cluster, genes related to degradation of aromatic compounds, *cox* genes contributing to energy production and a wealth of genes for sugar metabolism (Wagner-Döbler et al., 2010).

**TABLE 1:** Plasmids derived from NCBI database present in the plasmidome of Puquio de Campo Naranja.

**Plasmid**	**Host**	**Size (kb)**	**Isolation source**	**Number of contigs**	**NCBI accession number**	**References**
pDSHI01	*Dinoroseobacter shibae* DFL 12	191	Marine dinoflagellates from the Bay of Tokyo, Japan	3	NC_009955.1	Wagner-Döbler et al., 2010
pDSHI03	*D. shibae* DFL 12	126		1	NC_009957.1	Wagner-Döbler et al., 2010
pDSHI04	*D. shibae* DFL 12	86		1	NC_009958.1	Wagner-Döbler et al., 2010
pP73B	*Celeribacter indicus* P73^T^	136	Deep-sea sediment of the Indian Ocean	4	NZ_CP004395.1	Cao et al., 2015
pNS6001	*Confluentimicrobium* sp. EMB200-NS6	184	Oil- contaminated tidal flat	3	NZ_CP010870.1	NA
pNS6002	*Confluentimicrobium* sp. EMB200-NS6	157		2	NZ_CP010871.1	NA
pNS6003	*Confluentimicrobium* sp. EMB200-NS6	156		1	NZ_CP010872.1	NA

**NA, not available.**

Four contigs of the plasmidome matched with sequences of the plasmid pP73B from *Celeribacter indicus* P73^T^, a polycyclic aromatic hydrocarbon-degrading bacterium isolated from a deep-sea sediment of the Indian Ocean (Lai et al., 2014). Strikingly, Kothari et al. (2019) identified a plasmid from the groundwater plasmidome that reveals homology to a plasmid of *C. indicus* P73^T^ (pP73C, 122 kb). Matching sequences to plasmids pNS6001, pNS6002, and pNS6003 of *Confluentimicrobium* sp. EMB200-NS6 were also found. This alphaproteobacterium belongs to the Rhodobacteraceae and was isolated from an oil contaminated tidal flat (Tully et al., 2018).

All the above-mentioned plasmids were isolated from microorganisms thriving in saline environments but they cannot be considered as comparable due to the fact that the salinity in our system was much higher than in the other environments. Nevertheless, our results indicated the existence of plasmid sequences conserved in different environments.

### Replication and Mobilization Functions

Protein families covering putative gene products involved in plasmid replication and regulation are listed in Supplementary Table S2. Twenty-one of 32 known protein families were detected of which RHH_1 (PF01402.21), RepL (PF05732.11), and SSB (PF00436.25) were the most abundant. The RHH_1 family contains the plasmid-encoded transcriptional repressor CopG, previously referred to as RepA (Del Solar and Espinosa, 1992; Del Solar et al., 1995). This is the shortest transcriptional repressor reported to date and the first isolated from a plasmid. CopG represses its own synthesis and that of the replication initiator protein RepB. The RepL family covers replication initiation proteins RepL, which are involved in plasmid replication in Firmicutes, whereas SSB includes “single-stranded” binding proteins and also the primosomal replication protein N (PriB). Fourteen and 15 domains belonging to the major families of replication initiation proteins Rep_1 and Rep_3, respectively, were found.

Additionally, proteins containing domains related to plasmid mobilization, in particular MOB-type relaxases were searched (Supplementary Table S3). At least one representative from each of the nine different relaxase families was detected. The most abundant family was MOB_T_ for which the HMM profile (profile_MOBT) was recently built from an alignment of 498 relaxases (Garcillán-Barcia et al., 2020). Although mobilization elements have already been reported in previous plasmidomes (Schlüter et al., 2008; Kav et al., 2012; Sentchilo et al., 2013; Jørgensen et al., 2014), the classification in relaxase MOB families proposed by Garcillán-Barcia et al. (2009, 2020) was not performed in most of them. Meanwhile, Kothari et al. (2019) reported the MOB_Q_ and MOB_P_ families as the most abundant in circular plasmids from groundwater plasmidomes.

Thus, for the present plasmidome, the majority of the domains in putative plasmids are not closely related to the current database entries, but rather encode “as-yet-unknown” replication and mobilization systems, particularly since most of the microorganisms contributing to the plasmidome were not cultivable and live in a poorly studied environment. Furthermore, the diversity of replication proteins families suggests that the plasmidome harbors very different plasmids types.

### Virulence Factors and Metal-Resistance Genes

**FIGURE 5:**
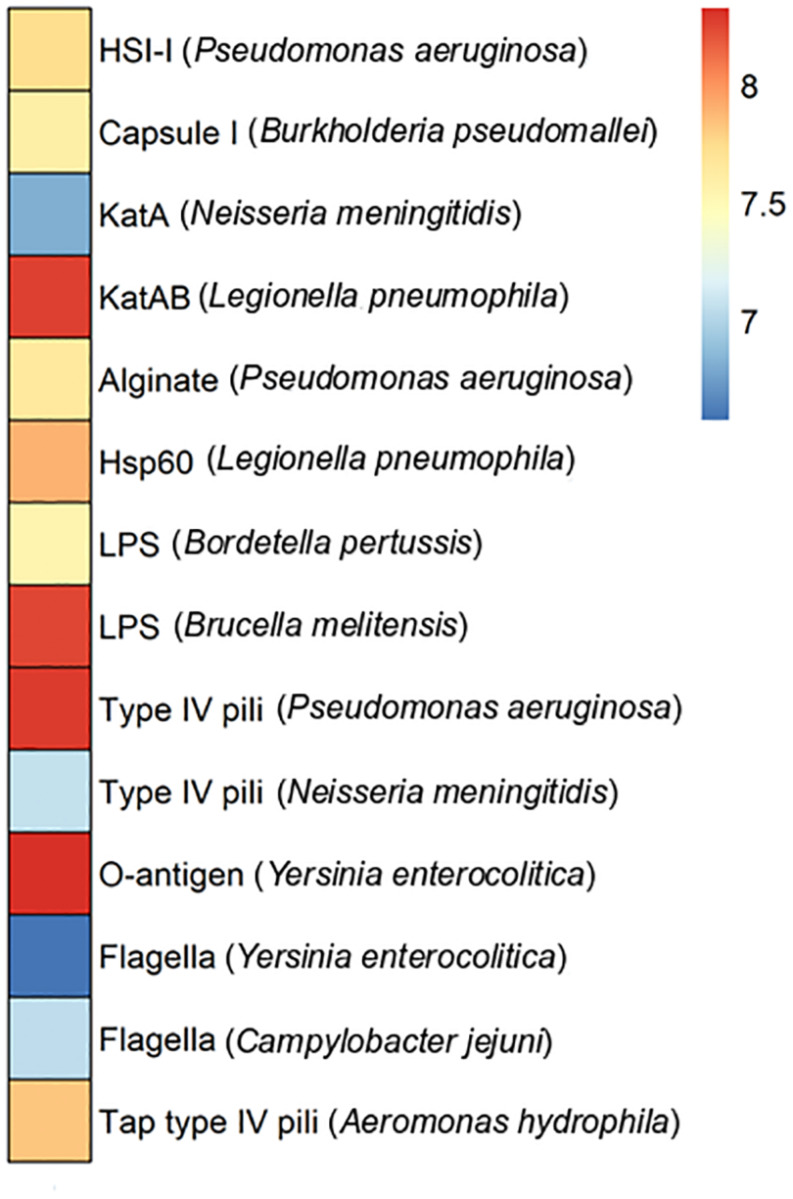
Virulence factors in the plasmidome of Puquio de Campo Naranja. The color scale indicates the abundance of each virulence factor in FPKM (fragments per kilobase per million). Values are plotted on a logarithmic scale. Pathogenic bacteria are specified in brackets.

Pathogenic bacteria infect hosts by employing virulence factors. In some cases, these factors are encoded at plasmid level. To assess plasmid-encoded virulence factors the Puquio de Campo Naranja plasmidome was analyzed through the VFDB database, which is an integrated and comprehensive resource for virulence factors of bacterial pathogens (Chen et al., 2005). Genes encoding KatAB and O-antigen virulence factors were the most abundant (Figure 5). The *katB* gene encodes a periplasmic catalase-peroxidase (KatB). The enzyme is important for intracellular survival and transmission of the pathogen *Legionella pneumophila*. O-antigen virulence factor includes the *gmd* gene encoding GDP-mannose 4,6-dehydratase, which is present in the pathogen *Yersinia enterocolitica.* This protein is involved in the LPS O-antigen biosynthesis pathway, which is part of bacterial outer membrane biogenesis. The absence of O-antigen in the outer membrane affects the ability to colonize the host and expression of other *Yersinia* virulence factors (Bengoechea et al., 2004). LPS virulence factor described in *Brucella melitensis* also exhibited a high relative abundance. The next most abundant putative genes encode type IV pili in *Pseudomonas aeruginosa.* These genes include the pilT gene encoding the twitching motility protein PilT, which attaches to host cells. Type IV pili are also involved in the biofilm formation. In addition, Carter et al. (2010) showed the importance of a type IV pilus for the horizontal gene transfer of the *P. aeruginosa* pathogenicity island PAPI-1, which is transferred by a conjugative mechanism. PAPI-1 is encoded by cluster comprising 10 genes, which are similar to the corresponding genes of the conjugative enterobacterial plasmid R64 (Kim and Komano, 1997).

Since a limited eukaryotic diversity was present in this extreme environment, it is not likely that DNA from pathogenic bacteria associated with large multicellular organisms is represented in the metagenome. Consequently, the occurrence and distribution of clinically relevant bacterial virulence genes across these natural (non-human) environments seem not to be significant or well-studied. Nevertheless, Søborg et al. (2016) also reported the occurrence of homologs to bacterial human virulence genes in metagenomic datasets of a variety of ecological niches, including a hypersaline mat from Guerrero Negro (Mexico), living microbialites from Cuatro Cienegas (Mexico) and a hot spring microbial mat from Yellowstone National Park (United States). Furthermore, phylogenetic analyses suggested that they represent ancient lineages in the phylogeny of the clinical genes (Søborg et al., 2016).

**FIGURE 6:**
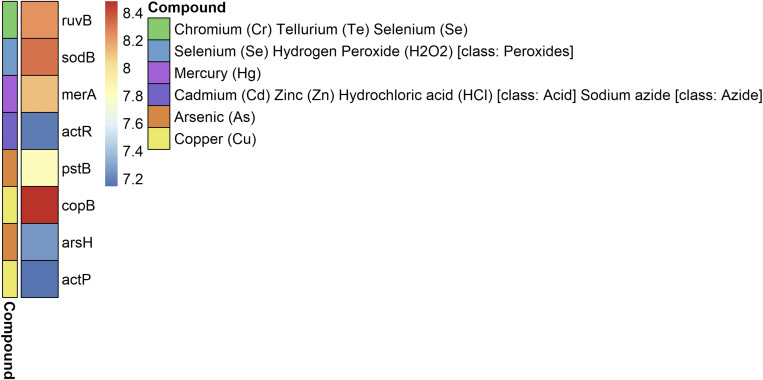
Metal-resistance genes in the plasmidome of Puquio de Campo Naranja. The color scale indicates the abundance of each gene in FPKM (fragments per kilobase per million). Values are plotted on a logarithmic scale. The other colors indicate the metal to which they confer resistance.

Genes associated with metals-resistance were also present in the plasmidome (Figure 6). The genes *copB* and *sodB* were the most abundant, followed by *ruvB* and *merA*. The *copB* gene was described in *Archaeoglobus fulgidus* and encodes an copper-transporting P-type ATPase, which is involved in copper efflux (Mana-Capelli et al., 2003). Although the *sodB* and *ruvB* genes are generally related to oxidative stress resistance and DNA repair, evidence of their participation in metal resistance has also been reported. The *sodB* gene codes for a Fe-dependent superoxide dismutase that destroys superoxide anion radicals, which are produced within the cells and are toxic to biological systems. The gene is also associated with resistance to selenium as superoxide dismutase activity is essential for the cellular defense against selenium salts (Bébien et al., 2002). The *ruvB* gene codes for an ATP-dependent DNA helicase that is involved in repairing DNA damage caused by chromate or its derivatives. In addition, it confers resistant to tellurite and selenite (Miranda et al., 2005). The *merA* gene was described in the plasmid pPB (80 kb) of a *Pseudomonas stutzeri* strain that degrades o-xylene (Baggi et al., 1987; Barbieri et al., 1989). In addition, *merA* gene was also encoded plasmid p5343 (8 kb) identified in a groundwater plasmidome by Kothari et al. (2019). MerA is mercuric reductase, which catalyzes the reduction of Hg^2+^ to volatile metallic mercury Hg^0^ (Reniero et al., 1995).

It is not the first time that metals-resistance genes have been reported in complex communities derived from extreme environments. In metagenomic studies of microbialites from Pavilion Lake (Canada), SEED functions and MetaCyc pathways related to heavy-metal detoxification were abundant. The occurrence of cobalt–zinc–cadmium resistance proteins, efflux pump proteins, phenylmercury acetate degradation, and chromate resistance was found (White et al., 2016). Subsequently, genome analysis of two bacteria isolated from these microbialites confirmed the presence of genes at chromosomal and plasmid level related to these pathways ([Bibr B91][Bibr B92]). Metagenomic analysis of hypersaline microbial mats and stromatolites from Shark Bay (Australia) also revealed functional annotations for a range of pathways involved in heavy-metal resistance, including those for zinc, chromium, mercury, copper, cadmium, and arsenic (Ruvindy et al., 2016). Arsenic metabolism genes are of particular interest in recent microbial mats because of a study providing evidence for arsenic cycling in fossil stromatolites (Sforna et al., 2014). Genes associated to arsenic metabolism were also present in Pavilion Lake microbialites (White et al., 2016). The stromatolite metagenome from Socompa Lake (Argentina) revealed a surprisingly diverse metabolic potential comprising all known types of arsenic resistance and energy generating pathways (Kurth et al., 2017). Furthermore, Sancho-Tomás et al. (2018) analyzed a living microbial mat from Laguna Brava (Chile) using geochemical techniques. The results indicated the occurrence of microbially-mediated arsenic cycling in these microbial mats.

The functional analysis of our plasmidome suggest the presence of diverse metal resistance gene clusters in the studied environment. The presence of genes encoding resistance to chromium, cadmium, arsenic and cooper could be explained by the high concentrations of the corresponding metals in the environment (Supplementary Table S1). However, putative genes encoding resistance to mercury were also detected despite of its low concentration (<0.1 mg Kg^–1^). Accordingly, Ruvindy et al. (2016) and White et al. (2016) reported similar findings at Pavilion Lake and Shark Bay, respectively. The idea that resistance to toxic metals emerged during early life on earth is in line with our results as the environment was polluted by volcanic and other geological activities (Silver and Phung, 1996). Nevertheless, the reason why resistance mechanisms are maintained in these microbialites is still unclear. A hypothesis is that antibiotic pressure helps maintaining the presence of metal resistance as they are both physically linked on plasmids (Foster, 1983). Moreover, White et al. (2018) have proposed the alternative hypothesis that initially these genes may have conferred heavy-metal resistance, but now they are operating under other stressors.

### Antibiotic Resistance Genes

**FIGURE 7:**
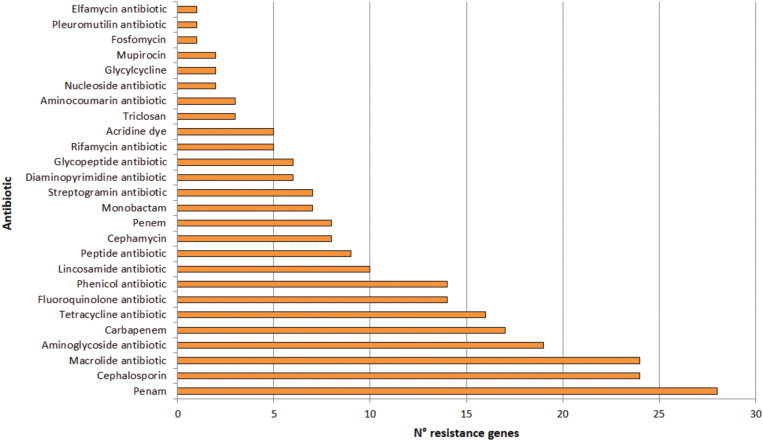
Number of genes conferring resistance to different types of antibiotics in the Puquio de Campo Naranja plasmidome. Putative antibiotic resistance genes were identified by the RGI software using the CARD database.

A total of 123 putative antibiotic resistance genes (ARGs) conferring resistance to 26 types of antibiotics were identified in the plasmidome (Figure 7 and Supplementary Table S4). Mainly, genes related to penam, cephalosporin and macrolide resistance were detected. Resistance genes to aminoglycosides, carbapenems, tetracyclines, fluoroquinolones, and phenicols were also present (Figure 7).

Similarly, Zhang et al. (2011) revealed high levels of known antibiotic resistances (35 ARGs) in the plasmidome of activated sludge from Shatin sewage treatment plant (Hongkong, China). ARGs coding for tetracycline (27.2%), macrolide (25.0%), and multidrug resistances (24.9%) were highly enriched, which could be expected in this human-impacted environment. Sentchilo et al. (2013) also studied the plasmid metagenome from microbial communities in two activated sludge systems, of which one receives mostly household wastewater and the other chemical industry wastewater. Both plasmidomes contained a wide variety of antibiotic resistance-encoding genes. In contrast, only little information on antibiotic resistances from remote and pristine environments is available. Van Goethem et al. (2018) recently described the presence of 177 ARGs in 17 remote and uncontaminated Antarctic surface soil metagenomes within the Mackay Glacier region of which most encoded single or multi-drug efflux pumps. The authors suggested that most ARGs likely represent functional efficient historical genes that have been vertically inherited over generations. In this study, the presence of ARGs in significant abundances in an environment without anthropogenic impact was also confirmed. Previously, resistance to various antibiotics in bacteria isolated from others high-altitude lakes of the Andean Puna was reported (Dib et al., 2008, 2009). In isolated strains from these lakes, a predominance of antibiotic resistances to azithromycin, erythromycin, clarithromycin, roxithromycin, streptomycin, chloramphenicol, gentamycin, kanamycin, tetracycline, and ampicillin was found, with unexpectedly high minimal inhibitory concentrations (MICs; >2 mg mL^–1^) for macrolides (Dib et al., 2008). In addition, experimental evidence for a plasmid-encoded resistance was also detected in an isolated actinobacterial strain (Dib et al., 2010a). Sequence analysis of linear plasmids revealed the presence of resistance genes (Dib et al., 2018). Accordingly, sequence analysis of the plasmidome also depicted that antibiotic resistances are widespread in the studied pristine environment in which antibiotic selective pressure is supposed to be absent. Nevertheless, it has been known that metal pressure can help retaining antibiotic resistance genes in bacteria (Baker-Austin et al., 2006) since these genes are generally linked to plasmids (Foster, 1983). That means antibiotic resistance genes encoded by the plasmidome along with metal resistances could be horizontally transferred between bacteria in the community. In addition, heavy metals have shown the ability to increase antibiotic resistance in bacteria independent of any antibiotic exposure (Nisanian et al., 2014).

Other studies of complex communities reported also genes related to antibiotic resistance. The metagenome of Pavilion Lake microbialite included genes encoding antibiotic resistance, i.e., beta-lactamases (White et al., 2016). Such results coincide with those obtained for our microbialite plasmidome (Figure 3). Moreover, Peimbert et al. (2012) detected pathways for producing antibiotics such as tetracycline, penicillin, streptomycin, novomycin, ansamycin, and vancomycin, as well as genes coding for beta-lactamases and metallo-lactamases in a microbial mat from Cuatro Ciénegas Basin (Mexico).

As these are environments are exposed to little or no human activity, it is possible that the presence of antibiotic resistance mechanisms is due to ecological factors rather than human influence. In fact, other functions in which antibiotics are also involved have been described. They can serve to compete with other organisms for survival or as a nutrient source (Dantas et al., 2008). In addition, they function as signaling molecules at low concentrations (Yim et al., 2007). Nevertheless, D’Costa et al. (2011) have shown that antibiotic resistance is a natural phenomenon that predates the selective pressure of clinical antibiotic use. For these reasons, extreme environments could constitute a reservoir of resistance genes and further studies in complex environmental communities could be clinically significant.

### Integrons, Transposons, and Insertion Sequences

Integrons are possible vehicles for distribution of antibiotic resistance genes. Integrons are probably not mobile by themselves, but they are often located in transposons, which in turn are found in conjugative plasmids involved in horizontal transfer (Sabaté and Prats, 2002). The presence of integrons was investigated in the plasmidome by comparing with the INTEGRALL database, but putative integrons were not detected. Furthermore, transposons were also not detected by employing the ISfinder platform. Nevertheless, 28 potential insertion sequences (IS) were found with IS*5*, IS*630*, and IS*4* as the most represented families (Supplementary Table S5).

It is well known that Tn*3* family transposons are one of the most abundant transposons in bacterial genomes that preferentially transpose into plasmid molecules (Szuplewska et al., 2015). It has to be noted that sequences associated with such transposons were not identified in the studied plasmidome. Among the reports on plasmidomes described to date, only a few studied the presence of these elements (Zhang et al., 2011; Jørgensen et al., 2014). In the case of Zhang et al. (2011), they reported the presence of only two IS associated with Tn*3* (IS*Sod9* and IS*Xc5*). Nevertheless, prior to plasmidome sequencing, they stated chromosomal DNA contamination, which was not completely removed. This fact, in addition to the origin of the sample used for the study of plasmids (sewage treatment plant), may be the reason for the differences existing with the present work. The absence of this type of elements or other similar ones could be explained by the unique characteristics of this microbial community within this environment, which might contain more unknown elements that have not been characterized.

### Phylogenetic Analysis

**FIGURE 8:**
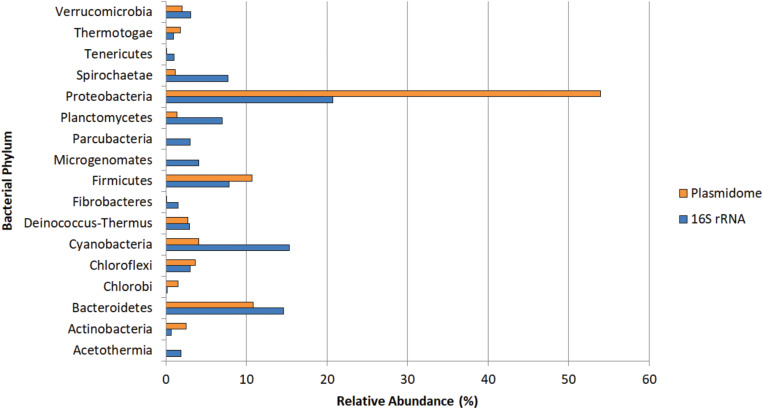
Phylogenetic analysis of the Puquio de Campo Naranja plasmidome at the phylum level. Orange bars show the relative abundance of each bacterial phylum by MG-RAST analysis using similarity to the RefSeq database with a maximum *E*-value of ≤ 10^– 5^. Blue bars show the relative abundance of each bacterial phylum by metagenomic DNA analysis using amplicon sequencing of 16S rRNA gene. Phyla that were less abundant than 1% in both datasets were not included.

The potential hosts of plasmids were estimated by phylogenetic assignment of the contigs. Approximately 95% of the contigs were assigned to the bacterial domain, slightly more than 4% to Archaea and the remaining to Eukaryota. In the bacterial domain, most of the contigs were assigned to Proteobacteria (54%), Bacteroidetes (10.8%), and Firmicutes (10.7%). The bacterial phyla distribution of plasmidome was compared with the phylogenetic analysis of the corresponding metagenomic DNA. In the metagenome, the phyla with the highest relative zOTU abundance were Proteobacteria (20%), Cyanobacteria (15%), and Bacteroidetes (14%) (Figure 8). The relative zOTU abundance of each bacterial class is shown in Supplementary Figure S1. Regarding Archaea domain, Euryarchaeota was the phylum with the highest zOTU abundance in the plasmidome and the metagenome (94.4 and 71.5%, respectively; Supplementary Figure S2). As mentioned by Kav et al. (2012), the difference in the phylogenetic distribution could be due to that certain phyla are hosting more plasmids than others, database bias (e.g., that a phylum is not well represented) or bias created by the plasmid extraction method used, which may favor the lysis of a certain group of microorganisms.

## Conclusion

For the first time, the structure and potential functions of the plasmidome present in the microbial community of a lake located in an extreme environment of the Puna were reported. This study showed that the generated genetic information for the plasmidome was different but with some overlap to the metagenome of the same environment. Thus, a plasmidome provides novel information, previously hidden by traditional metagenome-based studies. The study of the total plasmid population of this environment allowed the identification of a large proportion of genes encoding proteins with unknown function, supporting the idea that these pristine environments are a source of new biomolecules. Nevertheless, genetic features typically encoded by plasmids were found. One important feature were genes that permit microorganisms to survive under the harsh conditions of the studied environment. The abundant presence of genes that confer fitness under stress conditions could be due to the fact that a high number of copies of such genes provide a stronger phenotype. In addition, their plasmid location allows mobility and transfer within the microbial community.

Approximately 47% of the DNA metabolism genes were related to DNA repair functions. These functions might confer resistance or function as an adaptive mechanism to the high UV radiation exposure in the Andean Puna. Genes associated with metal resistance, mainly to cadmium, zinc, cobalt, copper, arsenic, mercury, chromium, and selenium were identified. Notably, antibiotic resistances were also widespread in the plasmidome of the studied pristine environment in which antibiotic selective pressure is supposed to be almost absent. Despite the differences of a pristine environment in the Andean Puna and an anthropogenically-impacted wastewater treatment plant environment, a certain degree of similarity with respect the proportion of antibiotic and metal resistance genes was recorded in both plasmidomes.

This research highlights the key role of plasmids for their hosts to thrive in a certain ecological niche. Moreover, it contributes to elucidate the potential ecological impact of extrachromosomal elements and their relationship to the distribution of resistance factors under the harsh conditions in these environments.

## Data Availability Statement

The sequence data have been deposited at ENA (European Nucleotide Archive) under accession numbers PRJEB34475 (plasmidome) and PRJEB30901 (metagenome).

## Author Contributions

JD and MP planned the work and drafted the manuscript. MP, DK, and GC performed the experiments and analyzed the data. MF and MS contributed to the lake samples for the study. RD, AP, and JD contributed to the conception and design and edited the manuscript. All authors interpreted the results and reviewed and revised the manuscript.

## Conflict of Interest

The authors declare that the research was conducted in the absence of any commercial or financial relationships that could be construed as a potential conflict of interest.

## Supplementary Material

FIGURE S1 Phylogenetic analysis of the Puquio de Campo Naranja plasmidome at bacterial class level.

FIGURE S2 Relative abundance of the archaeal phyla in the Puquio de Campo Naranja plasmidome.

TABLE S1 Physicochemical parameters measured in soil of Puquio de Campo Naranja.

TABLE S2 Plasmid replication-related Pfam entries in the Puquio de Campo Naranja plasmidome.

TABLE S3 Relaxase MOB families in the Puquio de Campo Naranja plasmidome.

TABLE S4 Putative antibiotic resistance genes in the Puquio de Campo Naranja plasmidome.

TABLE S5 Insertion sequences (IS) in the Puquio de Campo Naranja plasmidome.
