# Supplementary material for: First Report on the Plasmidome From a High-Altitude Lake of the Andean Puna
Source: Front Microbiol. 2020 Jun 23;11:1343. doi: 10.3389/fmicb.2020.01343 (PMC7324554; doi:10.3389/fmicb.2020.01343)
Supplement: FIGURE S1 — Phylogenetic analysis of the Puquio de Campo Naranja plasmidome at bacterial class level. [file Image_1.PDF]

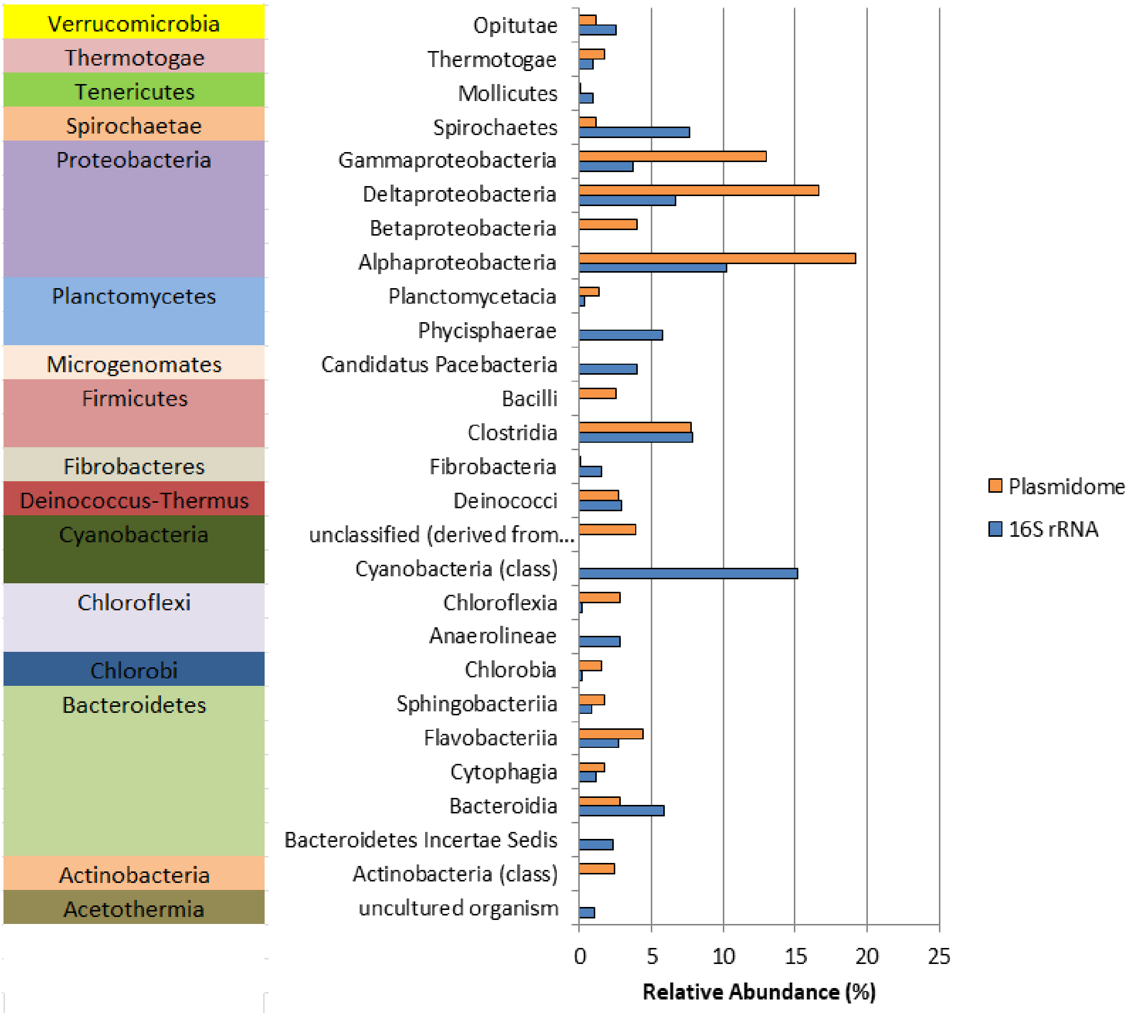

Supplementary Figure S1. Phylogenetic analysis of the Puquio de Campo Naranja plasmidome at class level. Orange bars show the relative abundance of each bacterial class by MG-RAST analysis using similarity to the RefSeq database with a maximum E-value of $\leq 10^{-5}$. Blue bars show the relative abundance of each bacterial class by metagenomics DNA analysis using amplicon sequencing of 16S rRNA gene. Classes with a relative abundance of less than 1% in both datasets were not included.
