# Supplementary material for: First Report on the Plasmidome From a High-Altitude Lake of the Andean Puna
Source: Front Microbiol. 2020 Jun 23;11:1343. doi: 10.3389/fmicb.2020.01343 (PMC7324554; doi:10.3389/fmicb.2020.01343)
Supplement: TABLE S1 — Physicochemical parameters measured in soil of Puquio de Campo Naranja. [file Table_1.PDF]

Supplementary Table S1. Physico-chemical parameters measured in soil of Puquio de Campo Naranja.

Parameter	Winter	Summer
pH relation 1:1 (UpH)	7.3	8.4
Salinity	Saline	Extremely saline
Electrical conductivity 1:5 (uS cm^{-1})	5,190	17,820
Humidity (% w/w)	59.5	20.8
Chloride soluble in aqueous medium (mg Kg^{-1})	19,700	30,545
Phosphate soluble in aqueous medium (mg Kg^{-1})	< 50.0	< 50.0
Nitrate soluble in aqueous medium (mg Kg^{-1})	90.0	< 50.0
Nitrite soluble in aqueous medium (mg Kg^{-1})	< 1.0	< 1.0
Ammoniacal Nitrogen (mg Kg^{-1})	400	30.0
Sulfate soluble in aqueous medium (mg Kg^{-1})	670	470
Arsenic (mg Kg^{-1})	109	82.2
Boron (mg Kg^{-1})	7,385	369
Calcium (mg Kg^{-1})	18,000	24,700
Lithium (mg Kg^{-1})	259	100.0
Total phosphorus (mg Kg^{-1})	370	115
Magnesium (mg Kg^{-1})	12,100	8,000
Total organic carbon (mg Kg^{-1})	9.5	2.1
Potassium (mg Kg^{-1})	12,700	1,660
Sodium (mg Kg^{-1})	77,200	29,200
Bromine (mg Kg^{-1})	< 50.0	< 50.0
Manganese (mg Kg^{-1})	4,430	44.9
Iron (mg Kg^{-1})	10,200	684
Total cooper (mg Kg^{-1})	28.3	< 5.0
Silica soluble in acidic medium (mg Kg^{-1})	2,810	6,090
Nickel (mg Kg^{-1})	3.7	< 0.6
Tin (mg Kg^{-1})	< 1.25	< 1.25
Mercury (mg Kg^{-1})	< 0.1	< 0.1
Lead (mg Kg^{-1})	4.7	< 0.6

Chromium (mg Kg^{-1})	51.3	2.7
Cadmium (mg Kg^{-1})	16.3	1.5
Aluminium (mg Kg^{-1})	1,240	617
Total cyanide (mg Kg^{-1})	< 0.25	< 0.25
