# Supplementary material for: First Report on the Plasmidome From a High-Altitude Lake of the Andean Puna
Source: Front Microbiol. 2020 Jun 23;11:1343. doi: 10.3389/fmicb.2020.01343 (PMC7324554; doi:10.3389/fmicb.2020.01343)
Supplement: TABLE S2 — Plasmid replication-related Pfam entries in the Puquio de Campo Naranja plasmidome. [file Table_2.PDF]

Supplementary Table S2. Plasmid replication-related Pfam in the Puquio de Campo Naranja plasmidome.

**Pfam name**	**Pfam code**	**Description**	**Hits**
Rep_1	PF01446.17	Replication protein	14
Rep_2	PF01719.17	Plasmid replication protein	0
Rep_3	PF01051.21	Initiator Replication protein	15
RepL	PF05732.11	Firmicute plasmid replication protein (RepL)	59
TrfA	PF07042.11	TrfA protein	6
RepA_C	PF04796.12	Plasmid encoded RepA protein	6
Rep_trans	PF02486.19	Replication initiation factor	5
RHH_1	PF01402.21	Ribbon-helix-helix protein, copG family	293
Rop	PF01815.16	Rop protein	0
RP-C	PF03428.13	Replication protein C N-terminal domain	13
RPA	PF10134.9	Replication initiator protein A	12
RepA_N	PF06970.11	Replication initiator protein A (RepA) N-terminus	0
RepC	PF06504.11	Replication protein C (RepC)	4
Replicase	PF03090.17	Replicase family	0
IncFII_repA	PF02387.15	IncFII RepA protein family	0
PriCT_1	PF08708.11	Primase C terminal 1 (PriCT-1)	3
DUF1424	PF07232.11	Putative rep protein (DUF1424)	1
Phage_CRI	PF05144.14	Phage replication protein CRI	4
PriCT_2	PF08707.11	Primase C terminal 2 (PriCT-2)	0
Prim-Pol	PF09250.11	Bifunctional DNA primase/polymerase, N-terminal	0
SSB	PF00436.25	Single-strand binding protein family	45
RepA1_leader	PF08048.12	Tap RepA1 leader peptide	0
DUF1738	PF08401.11	Domain of unknown function (DUF1738)	3
UvrD-helicase	PF00580.21	UvrD/REP helicase N-terminal domain	29
RepB-RCR_reg	PF10723.9	Replication regulatory protein RepB	10
Replic_Relax	PF13814.6	Replication-relaxation	24
KorB_C	PF06613.11	KorB C-terminal beta-barrel domain	0
KorB	PF08535.10	KorB domain	39
Activator-TraM	PF11657.8	Transcriptional activator TraM	2
pRN1_helical	PF13010.6	Primase helical domain	0
KORA	PF16509.5	TrfB plasmid transcriptional repressor	19
Rol_Rep_N	PF18106.1	Rolling Circle replication initiation protein N-terminal domain	0
