# Supplementary material for: First Report on the Plasmidome From a High-Altitude Lake of the Andean Puna
Source: Front Microbiol. 2020 Jun 23;11:1343. doi: 10.3389/fmicb.2020.01343 (PMC7324554; doi:10.3389/fmicb.2020.01343)
Supplement: TABLE S3 — Relaxase MOB families in the Puquio de Campo Naranja plasmidome. [file Table_3.PDF]

Supplementary Table S3. Relaxase MOB families in the Puquio de Campo Naranja plasmidome.

**Relaxase MOB family**	**Profile HMM**	**Hits**
MOB_B	T4SS_MOBB	1
MOB_C	T4SS_MOBC	4
MOB_F	profile_MOBF	3
MOB_H	T4SS_MOBH	4
MOB_P	T4SS_MOBP1	5
	T4SS_MOBP2	0
	T4SS_MOBP3	1
MOB_Q	T4SS_MOBQ	5
MOB_T	profile_MOBT	29
MOB_V	T4SS_MOBV	3
MOB_M	profile_MOBM	6
