# Supplementary material for: First Report on the Plasmidome From a High-Altitude Lake of the Andean Puna
Source: Front Microbiol. 2020 Jun 23;11:1343. doi: 10.3389/fmicb.2020.01343 (PMC7324554; doi:10.3389/fmicb.2020.01343)
Supplement: TABLE S5 — Insertion sequences (IS) in the Puquio de Campo Naranja plasmidome. [file Table_5.PDF]

Supplementary Table S5. Insertion sequences (IS) in the Puquio de Campo Naranja plasmidome.

IS	Family	Group	Origin	Length	IR	DR	ORF	Accession Number
ISAcma1	IS6		*Acaryochloris marina*	846	14/15		236 (79-789)	NC_009932
ISAcma10	ISL3		*A.marina*	1385	25/26	8	414 (100-1344)	NC_009925
ISAcma11	IS4	ISPepr1	*A. marina*	1347	19	6	322 (362-1330)	NC_009925
ISAcma12	ISAs1		*A. marina*	166	16/17	10	190 (67-639)<br>191 (581-1156)<br>362 (67-1156)	NC_009925
ISAcma13	IS3	IS150	*A. marina*	1263	22/25	3	101 (68-373)<br>319 (265-1224)<br>386 (68-1224)	NC_009925
ISAcma14	IS630		*A .marina*	1138	15/28	4	369 (27-1136)	NC_009925
ISAcma15	IS630		*A. marina*	1083	22/29	2	334 (74-1078)	NC_009925
ISAcma16	IS4	IS4	*A. marina*	1483	18	10	472 (42-1460)	NC_009925
ISAcma17	IS5	IS5	*A. marina*	1697	13/17	6	526 (117-1697)	NC_009925
ISAcma18	IS4	IS50	*A. marina*	1530	16/19	10	405 (300-1517)	NC_009925
ISAcma19	IS4	IS10	*A. marina*	1316	15/17	9	388 (103-1269)	NC_009925
ISAcma41	IS630		*A. marina*	1139	14/28	4	365 (40-1137)	NC_009925
ISAcma42	IS701	ISAba11	*A. marina*	1140	18/19	5	340 (73-1095)	NC_009925
ISAtsp5	IS4	IS10	*Arthrospira* sp.	1320		9	398 (104-1300)	CP028914.1
ISCph12	IS5	IS5	*Chlorobium phaeobacteroides*	1635	15/16		498 (124-1619)	NC_010831
ISCwa2	IS5	IS5	*Crocosphaera watsonii*	1691	14/15		508 (137-1663)	NZ_AADV02000002
ISCysp15	IS5	IS5	*Cyanothece* sp.	1690	15/16	6	508 (136-1662)	NC_011726
ISLca2	IS5	IS5	*Lactobacillus casei*	1563	13/20	6	471 (136-1551)	NC_010999
ISMae24	IS630		*Microcystis aeruginosa*	1084	19/23	2	332 (81-1079)	NC_010296
ISMae6	IS5	IS5	*M. aeruginosa*	1682	16/18	6	498 (136-1632)	NC_010296
ISMafe1	IS5	IS5	*Mariprofundus ferrooxydans*	1745	12/15	6	548 (77-1723)	NZ_AATS01000020

ISPaen2	IS5	IS5	*Paenibacillus* sp.	1786	15/17	6	544 (133-1767)	NZ_GG695980
ISPeth2	IS5	IS5	*Pelotomaculum thermopropionicum*	1642	13/14	8	493 (122-1603)	NC_009454
ISPsa2	IS6		*Piscirickettsia salmonis*	863	17/19		242 (69-797)	HM563000
ISSysp4	IS5	IS5	*Synechococcus* sp.	1724	12/14	6	513 (117-1658)	NZ_AANO01000005
ISTer1	IS630		*Trichodesmium erythraeum*	942	21/23	4	119 (55-414)<br>179 (362-901)<br>281 (55-901)	NC_008312
ISVsa8	IS630		*Aliivibrio salmonicida*	1122	21/29		140 (109-531)<br>211 (480-1115)<br>335 (109-1115)	NC_011312
ISVsp12	IS630		*Verrucomicrobium spinosum*	1125	25/28	2	345 (76-1113)	NZ_ABIZ00000000

IR: inverted repeat sequences

DR: direct repeat sequences

ORF: open reading frame
